# Effects of Carbon/Nitrogen Ratio on Growth, Intestinal Microbiota and Metabolome of Shrimp (*Litopenaeus vannamei*)

**DOI:** 10.3389/fmicb.2020.00652

**Published:** 2020-04-15

**Authors:** Haipeng Guo, Lei Huang, Songtao Hu, Chen Chen, Xiaolin Huang, Wei Liu, Sipeng Wang, Yueyue Zhu, Yueji Zhao, Demin Zhang

**Affiliations:** ^1^State Key Laboratory for Managing Biotic and Chemical Threats to the Quality and Safety of Agro-Products, Ningbo University, Ningbo, China; ^2^School of Marine Sciences, Ningbo University, Ningbo, China; ^3^Zhejiang Mariculture Research Institute, Wenzhou, China

**Keywords:** C/N ratio, shrimp, biofloc formation, intestinal microbiota, intestinal metabolome

## Abstract

Increasing the C/N ratio of input feed has been reported as a practical approach for improving water quality and enhancing shrimp growth through changing the bacterial community of rearing water. However, little is known about the effects of different C/N ratios of feed input on the intestinal microbiota and metabolome of shrimp. In the present study, the effects of three different C/N ratio levels (CN6, CN10, and CN15) maintained by adding sucrose on the growth, intestinal microbiota and metabolome of *Litopenaeus vannamei*, and bioflocs formation were investigated after 17 days of feeding. The results indicated that higher C/N ratio (10 and 15), especially CN15, of feed input significantly enhance the length and weight of shrimp individuals accompanied by a significant accumulation of bioflocs, compared to that of CN6. The increase of C/N ratio input decreased the α-diversity of the intestinal microbiota and changed the microbial community structure through increasing the relative abundance of Actinobacteria, Rhodobacteraceae (mainly consist of *Roseobacter* and *Paracoccus* groups), Alteromonadaceae, and inhibiting the growth of Cyanobacteria, certain Rhodobacteraceae, Mycoplasmataceae and *Vibrio*. The change of microbial community caused by increasing C/N ratio input was closely associated with various bioactive metabolites of flavonoids, benzenoids, prenol lipids, and indole derivatives, which are benefit for shrimp growth either as an antimicrobial agent or as a nutrient component. Overall, this study demonstrated that manipulating high C/N ratio of feed input helps to the growth of shrimp through increasing the relative abundance of potential beneficial bacteria and the accumulation of various bioactive metabolites to suppress the growth of detrimental bacteria.

## Introduction

For decades, shrimp farming has been expanding, and become one of the main mariculture industries of China. *Litopenaeus vananmei* is one of the dominant species used for shrimp culture (Argue et al., 2002). However, the aquaculture industry of *L. vananmei* has encountered a variety of problems, such as germplasm degradation, disease outbreaks and water quality deterioration, which have seriously hindered its further development (Bachère, 2000; Thitamadee et al., 2016). To solve these problems, various chemicals like disinfectants and antibiotics have commonly been used in shrimp farming to control the occurrence of diseases, but the abuse of these drugs will lead to the antibiotic resistance of bacterial pathogens and toxic chemical residues in shrimp, which may pose a threat to human health (Costa et al., 2015; Zhao et al., 2018). In addition, the large amount of water exchanges in the process of aquaculture not only causes nutrient loss but also results in serious pollution to the surrounding environment (Bachère, 2000). Therefore, an eco-friendly aquaculture model and practical technologies are urgently necessary for controlling shrimp diseases and guaranteeing sustainable development of shrimp-culture industry (Kumar et al., 2016).

Recently, biofloc technology (BFT) has been successfully used in high-density and zero-exchange aquaculture of shrimp due to its significant advantages in improving the water quality of aquaculture and increasing the yield of shrimp (Crab et al., 2012). Bioflocs participate in the process of inorganic nitrogen assimilation by heterotrophic bacteria, which can be stimulated to grow by increasing the carbon/nitrogen (C/N) ratio in the water (Avnimelech, 1999). It has been reported that increasing the C/N ratio of culture systems by adding an external carbon source could significantly change the diversity and community structure of the heterotrophic microorganisms in the aquaculture water (Panigrahi et al., 2018). The heterotrophic bacteria could assimilate ammonia nitrogen and convert it into microbial protein, thus not only improving the water quality but also providing necessary nutrient for the growth of shrimp (Wei et al., 2016). In addition, many researchers have reported that biofloc application can also increase the feed utilization, improve the innate immunity and antioxidant capacity of shrimp, and enhance the resistance against pathogens (Xu and Pan, 2013; Julie et al., 2014). However, the mechanisms of biofloc application in improving the growth and health of shrimp are still limited.

Intestinal microbiota has been reported to play an important role in maintaining intestinal homeostasis (Chung and Kasper, 2010). This homeostasis is closely associated with a range of biological processes of host, such as digestion, innate immunity, against pathogens and functional maturation of the gut in various animals (Peterson and David, 2014). The growth of aquatic animals highly depends on the water environment, and thus the aquatic microorganisms likely affect the microbial colonization in intestine of aquatic animals (Giatsis et al., 2015). In addition, microbial bioflocs including numerous heterotrophic microbes are likely ingested by shrimp, and certain bacteria may also colonize the intestine (Xu et al., 2016). The change of intestinal microbiota will inevitably affect the production of metabolites, which may further cause the changes in the physiological function and immunity system of shrimp (Levy et al., 2017). Therefore, the intestinal microbiota and metabolome can be used to evaluate the effects of biofloc application on the growth and health of shrimp. This study aims to investigate the effects of three different C/N ratios regulated by adding the sucrose on the growth, intestinal microbiota and metabolome of shrimp (*Litopenaeus vannamei*). In addition, the crosstalk of key OTUs and differential metabolites was showed by a network analysis.

## Materials and Methods

### Experimental Design and Sample Collection

The experiment was carried out from June 10 to 26, 2017 at the Yongxing Base of Zhejiang Mariculture Research Institute in Wenzhou, Zhejiang Province, China. Prior to the experiment, 210 healthy juvenile shrimps of similar size (4.34 ± 0.64 g) obtained from Zhejiang Mariculture Research Institute were randomly stocked in each of the fifteen experimental tanks with a capacity of 600 L. These tanks were randomly divided into three groups (5 replicates per group): CN6 (C/N ratio is about 6), feeding basal diet (44.20% w/w carbon, 7.04% w/w Nitrogen; Alpha Feed Co., Ltd., Shenzhen, China); CN10, feeding the mixture of sucrose (42.1% carbon, purity 99%; Sinopharm Chemical Reagent Co., Ltd., Shanghai, China) and basal diet with the ratio of 3:5 to obtain a C/N ratio of about 10:1; CN15, feeding the mixture of sucrose and basal diet with the ratio of 3:2 to obtain a C/N ratio of about 15:1. The feeding trial lasted for 17 days. During the experiment, the above diets were added with a rate of 5% of shrimp weight three times per day (07:00 am, 12:00 pm, and 17:00 pm). The water was not changed for the first week of the experiment, and then it was changed by 1% every day after 1 week. At the beginning (Day 0, named as Original samples) and the end of the experiment (Day 17), the intestine of five shrimps from each tank were randomly sampled and equally divided into two parts under liquid nitrogen. One part was stored at −80°C for 16S rRNA amplicon sequencing and the other part was lyophilized for metabolomics analysis.

### Assessment of Growth Parameters and Biofloc Contents

Growth parameters such as the length and weight of shrimp individuals were measured at 0, 11, and 17 days of feeding. For each group, 10 shrimp individuals were randomly harvested from each tank to measure the individual length and individual weight. The shrimp length was measured on a centimeter scale and the individual weight was recorded by an electronic balance after drying with clean paper. The bioflocs volume was tested by using an Imhoff cone at 11 and 17 days of feeding. Briefly, 1 L of the culture water from each tank was taken into the Imhoff cone and left to stand for 30 min. Then the volume of solids at the bottom of the cone was measured in ml L^–1^.

### DNA Extraction

Totally, 20 gut samples (5 for each group) were obtained. Intestinal bacterial DNA was extracted by using a QIAamp DNA Stool mini kit (Qiagen, GmbH, Hilden, Germany) according to the manufacturer’s instructions. DNA concentrations were quantified using a Nanodrop 2000 instrument (Thermo Fisher Scientific, Wilmington, DE, United States) and then frozen at −80°C prior to amplification.

### Bacterial 16S rRNA Gene Amplification and MiSeq Sequencing

The V4 region of the bacterial 16S rRNA gene was amplified through polymerase chain reaction (PCR) using the following primers: 338F (5′-ACTCCTACGGGAGGCAGCAG-3′) and 806R (5′-GGACTACHVGGGTWTCTAAT-3′). PCR products for each sample were pooled and purified using a PCR fragment purification kit (TaKaRa Biotech, Japan). The amplicon products were sequenced using an Illumina MiSeq platform (Illumina, SanDiego, CA, United States).

### Processing of Illumina Sequencing Data

Raw FASTQ files were processed using the Quantitative Insights Into Microbial Ecology (QIIME v1.9.1) (Caporaso et al., 2010). Overlapping reads were merged using FLASH with default parameters (Tanja and Salzberg, 2011). Chimeras in the remaining sequences were identified with the USEARCH v6.1 (Edgar et al., 2011) and binned into operational taxonomic units (OTUs, 97% similarity level) using UCLUST (Edgar, 2010). OTUs affiliated with Archaea, chloroplast, mitochondrion, unassigned, and unclassified sequences and singletons were removed from the data before downstream analysis. The numbers of final clean reads in each sample were ranged from 33,390 to 58,142 reads.

The α-diversity parameters (richness, Shannon, phylogenetic diversity and evenness) were calculated using QIIME and visualized using the ggplot2 package in R environment. The principal coordinate analysis (PCoA) and Permutational multivariate analysis of variance (PERMANOVA) based on Bray-Curtis dissimilarity were performed to evaluate the overall differences in bacterial communities among groups. The microbiome composition was analyzed at the class (relative abundance of >1%) and family (relative abundance of >5%) levels. The top 40 abundant OTUs were selected for study of the shifts of bacterial community compositions in intestines at distinct experimental conditions, and the abundance of each OTU was scaled by color in a heat map using the pheatmap package.

### Metabolomics Analysis

The dry intestinal content powers (15 mg) were homogenized in the extraction solvent containing 600 μl of methanol-water (4:1, v/v) and 20 μl of internal standard (0.3 mg mL^–1^l-2-chlorophenylalanine in methanol). Then the mixture was settled at −20°C for 30 min after extracting with ultrasonic in an ice bath for 10 min. Finally, the samples were centrifuged at 4°C and 13000 rpm for 15 min and the supernatant was used for LC-MS analysis. LC-MS was performed on a Waters UPLC I-class system equipped with a binary solvent delivery manager and a sample manager, coupled with a Waters VION IMS Q-TOF Mass Spectrometer equipped with an electrospray interface (Waters Corporation, Milford, United States). LC Conditions: Column: Acquity BEH C18 column (100 mm × 2.1 mm i.d., 1.7 μm; Waters, Milford, United States). Solvent: The column was maintained at 45°C and separation was achieved using the following gradient: 5–20% B over 0–2 min, 20–60% B over 2–8 min, 60–100% B over 8–12 min, the composition was held at 100% B for 2 min, then 14–14.5 min, 100% to 5% B, and 14.5–15.5 min holding at 5% B at a flow rate of 0.40 ml min^–1^, where B is acetonitrile (0.1% (v/v) formic acid). Injection Volume was 3.00 μL and Column Temperature was set at 45.0°C. The mass spectrometric data was collected using a Waters VION IMS Q-TOF Mass Spectrometer equipped with an electrospray ionization (ESI) source operating in either positive or negative ion mode. The source temperature and desolvation temperature were set at 120°C and 500°C, respectively, with a desolvation gas flow of 900 L h^–1^. Centroid data was collected from 50 to 1,000 m/z with a scan time of 0.1 s and interscan delay of 0.02 s over a 13 min analysis time. QC sample was prepared by mixing aliquots of the all samples to be a pooled sample, and then analyzed using the same method with the analytic samples. The QCs were injected at regular intervals (every eight samples) throughout the analytical run to provide a set of data from which repeatability can be assessed. Before the pattern recognition, the original data was filtered, identified, integrated, retained time correction, aligned and normalized by the metabolomics processing software progenesis QI (Waters Corporation, Milford, United States), which was built in the instrument. Finally, a data matrix of retained time, mass to charge ratio and peak strength were obtained.

Normalized LC-MS data was employed for all downstream analyses. The progenesis QI software was applied to screening for differential metabolites between groups (VIP > 1, *P* < 0.05) using multidimensional analysis (OPLS-DA). The software was set to automatically search multiple database searched such as Human Metabolome Database (HMDB)^1^ and LIPID MAPS^2^. The Venn diagram was applied to analyze the shared and specific metabolites with significant difference between groups by using the R software. The principal component analysis (PCA) based on Euclidean distances was applied to evaluate the overall differences in the composition of metabolites among different groups. The network between OTU abundances and metabolite contents was drawn with Cytoscape 3.4 (Stefanini et al., 2017). Statistical analysis was carried out by one-way analysis of variance using SPSS (SPSS Inc., United States, version 18.0).

## Results

### Effects of Different C/N Ratios on Shrimp Growth and Biofloc Contents

**FIGURE 1:**
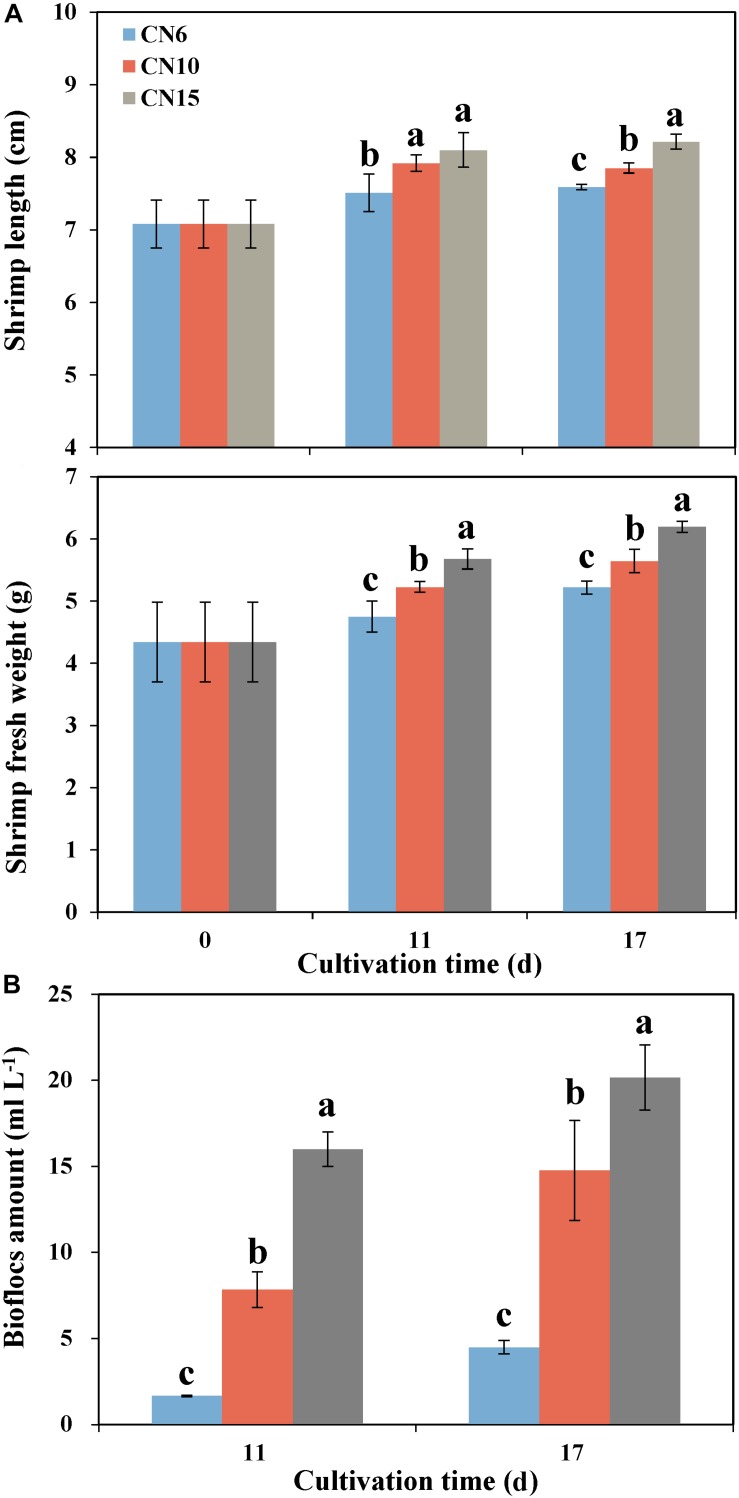
The length and weight of shrimp individuals **(A)** and bioflocs **(B)** volume in different experiment groups (CN6, CN10, and CN15) after 11 and 17 days of experiment. Data present means ± deviation (*n* = 10). Different lowercase letters on the histogram indicate significant difference (one-way ANOVA, *P* < 0.05).

Increasing the C/N ratios significantly improved the growth parameters of shrimp such as the individual length and individual weight, compared to that of the CN6 (Figure 1A). Among these groups, the CN15 had the best growth performance of shrimp, followed by the CN10. The average individual lengths of CN15 were 7.8 and 8.3% higher than that of CN6 after 11 and 17 days of feeding, respectively, and correspondingly the increment ratios of individual weights were 19.5 and 18.7%, respectively (Figure 1A). Increment of C/N ratio markedly accelerate the accumulation of bioflocs (Figure 1B). The bioflocs volumes in groups CN6, CN10, and CN15 were 1.7, 7.8, and 16.0 ml L^–1^ after 11 days of feeding, respectively, and correspondingly they were 4.5, 14.8, and 20.2 ml L^–1^ after 17 days of feeding, respectively (Figure 1B).

### Changes in the Gut Microbiome of Shrimp Supplied With Different C/N Ratios

**FIGURE 2:**
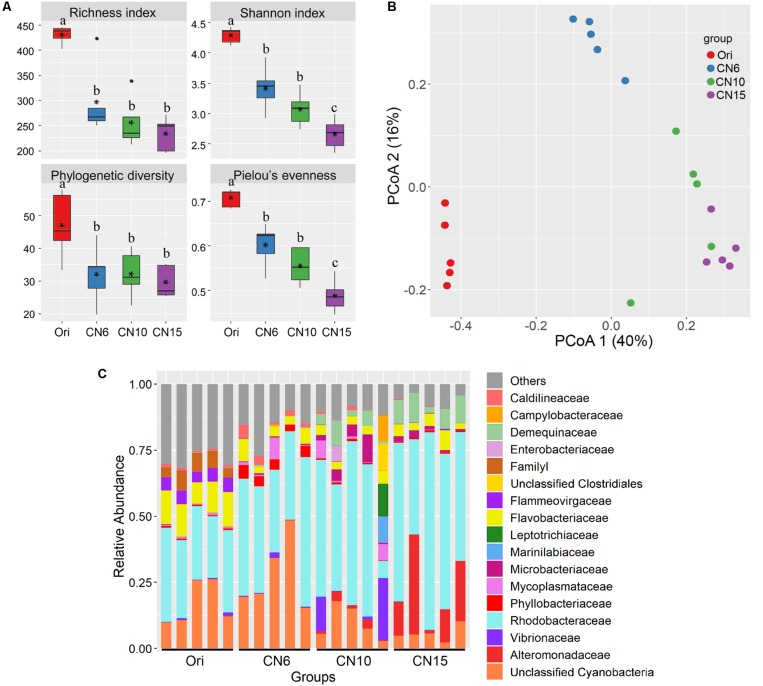
**(A)** Differences in intestinal microbial α-diversity indexes among the four groups. Different lowercase letters on the histogram indicate significant difference (one-way ANOVA, *P* < 0.05); **(B)** Principal coordinate analysis (PCoA) visualized the differences of intestinal microbial community structure among different groups; **(C)** Relative abundances of the dominant bacterial families (relative abundance >5%) in the shrimp intestines.

The four alpha-diversity indices including richness, Shannon, phylogenetic diversity and evenness at day 0 were significantly higher than those at day 17 (Figure 2A). Among the three different C/N ratio groups, these alpha-diversity indices, especially with the Shannon index and evenness, showed a decreasing trend as the C/N ratio increased (Figure 2A). In addition, a PCoA analysis was carried out to further evaluate the dissimilarity of bacterial composition among the four groups. The results showed a distinct dissimilarity in the bacterial community among the four groups (Figure 2B). This pattern was further verified by the PERMANOVA analysis, which indicated that the bacterial communities among different groups were significantly different (*P* < 0.05) (Table 1).

**TABLE 1:** Permutational multivariate analysis of variance (PERMANOVA) based on Bray-Curtis dissimilarity was used to test the differences of shrimp gut microbiota among different groups (999 permutations).

	F	*P*
Ori vs. CN6	17.539	0.014
Ori vs. CN10	10.858	0.009
Ori vs. CN15	27.045	0.012
CN6 vs. CN10	4.060	0.010
CN6 vs. CN115	10.526	0.012
CN10 vs. CN115	2.961	0.008

*Ori: the original samples (Day 0).*

Moreover, the relative abundance of the dominant classes (relative abundance >1% at least in one group) also markedly differed among the four groups (Supplementary Figure S1). The relative abundances of Alphaproteobacteria, Actinobacteria and Bacilli were significantly higher in groups CN10 and CN15 than those in group CN6, while the Cyanobacteria exhibited an opposite trend. The relative abundance of Gammaproteobacteria in group CN15 was significantly higher than that in the other groups. In addition, a number of bacteria belonged to Cytophagia and Verrucomicrobiae were found at day 0 (Ori), while they almost disappeared at the day 17 (Supplementary Figure S1). Similarly, there were remarkable differences in bacterial composition among different groups at the family level (Figure 2C). The relative abundances of Rhodobacteraceae, Alteromonadaceae and Demequinaceae were markedly increased at the high C/N ratios levels, especially with group CN15, compared to those in the group CN6; while the unclassified Cyanobacteria was overwhelmed in the groups CN6 and Ori (Figure 2C).

**FIGURE 3:**
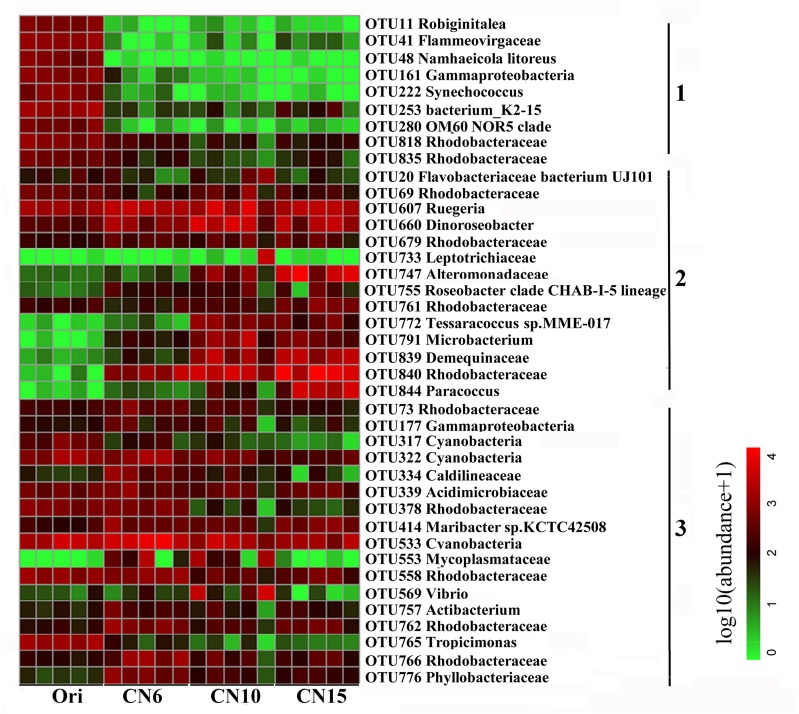
Heatmap summarizing the relative abundance of the top 40 abundant OTUs in the intestine of *L. vannamei*. Microbial abundance was scaled with log transformation in the heatmap. The OTUs were organized by their phylogenetic positions; the taxa of OTUs are shown on the right. 1, cluster 1 indicating that the abundance was the highest in the group Ori (Day 0); 2, cluster 2 indicating that the abundance was increased in the group CN10, CN15 or both of them, compared to that in the group CN6; 3, cluster 3 indicating that the abundance was decreased in the group CN10, CN15 or both of them, compared to that in the group CN6.

**FIGURE 4:**
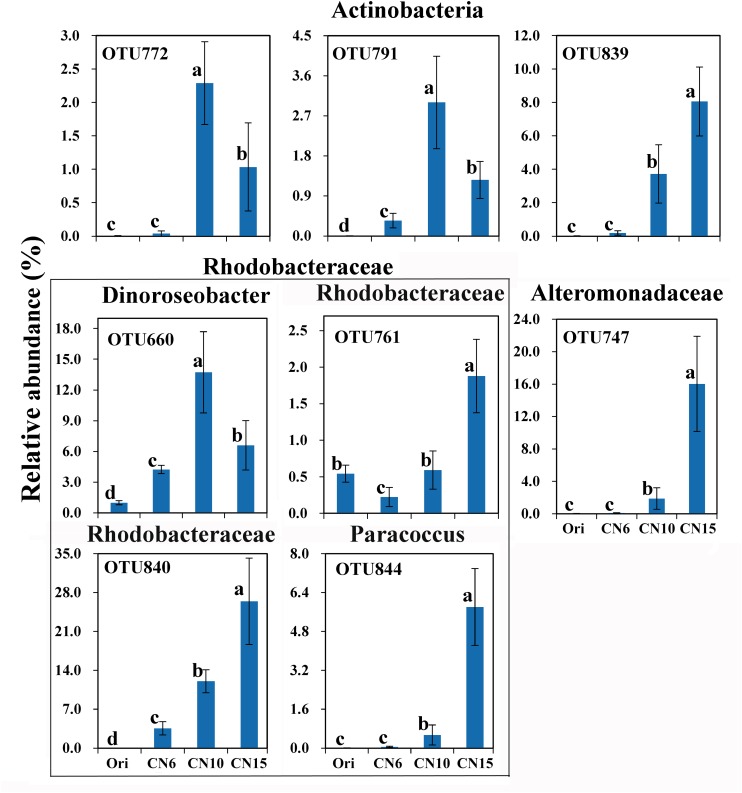
The significantly improved OTUs in average relative abundance at the CN10 and CN15 compared to the CN6. The detailed information of indicator taxa (OTUs) was showed in Supplementary Table S1. Values represent mean ± standard deviation (*n* = 5). Bars with different letters indicate significant differences at *P* < 0.05 according to Duncan’s multiple range tests.

**FIGURE 5:**
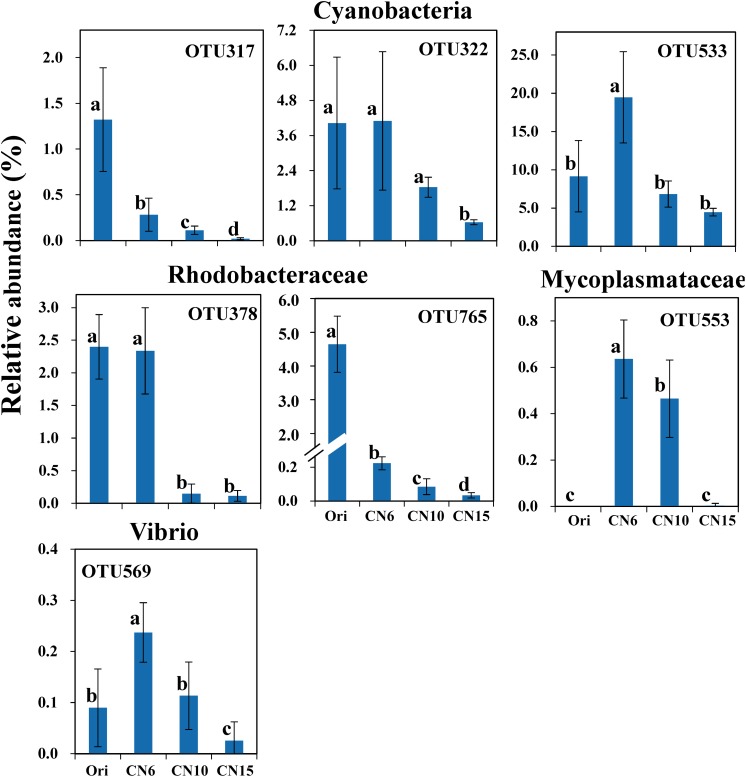
The significantly reduced OTUs in average relative abundance at the CN10 and CN15 compared to the CN6. The detailed information of indicator taxa (OTUs) was showed in Supplementary Table S1. Values represent mean ± standard deviation (*n* = 5). Bars with different letters indicate significant differences at *P* < 0.05 according to Duncan’s multiple range tests.

To further evaluate the discriminatory taxa among different groups, the relative abundance of the top 40 abundant OTUs was visualized by a heapmap. The result showed that the top 40 abundant OTUs were divided into 3 clusters according to the difference in relative abundance among different groups (Independent *t*-test, *P* < 0.05). The cluster 1 included 9 OTUs, the abundances of which were the highest at day 0 (Ori) and showed no significant difference among the three C/N ratios groups (Figure 3). The cluster 2 consisted of 14 OTUs that were enriched in the high C/N ratios groups (CN10, CN15), especially the OTUs belonging to Actinobactria (OTU772, 791 and 839), Rhodobacteraceae (OTU69, 607, 660, 679, 755, 761, 840, and 844) and Alteromonadanceae (OTU747), the relative abundances of which were 3.4-59.3 and 2.2-228.3-fold higher in groups CN10 and CN15 than that in group CN6, respectively (Figures 3, 4). The cluster 3 included 17 OTUs that were underrepresented in the CN10 and CN15, compared to those in CN6. For example, the relative abundances of OTU317, 322 and 533 in group CN10 were significantly decreased by 60.6, 55.3, and 64.9%, respectively, and the corresponding decreases in group CN15 were 93.2, 84.6, and 77.0%, respectively, compared to those in the group CN6 (Figures 3, 4). The relative abundances of OTU378 in group CN6 were 15.7-, 20.4-fold higher than that in groups CN10 and CN15, respectively. In addition, the abundances of OTUs belonging to Mycoplasmataceae (OTU553) and *Vibrio* (OTU569) were significantly reduced by 99.3 and 89.3% in the group CN15, respectively, compared to those in the group CN6 (Figures 3,  5).

### Changes in the Gut Metabolome of Shrimp Supplied With Different C/N Ratios

**FIGURE 6:**
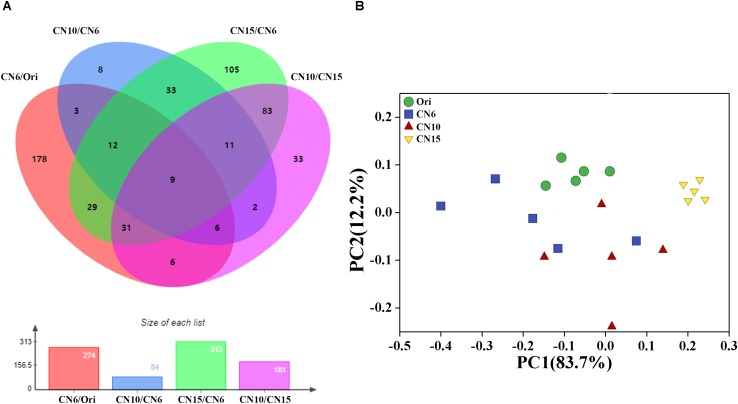
**(A)** Venn diagram analysis of differential metabolites screened by multidimensional analysis (OPLS-DA) among different groups; **(B)** Principal component analysis of the gut metabolome.

**FIGURE 7:**
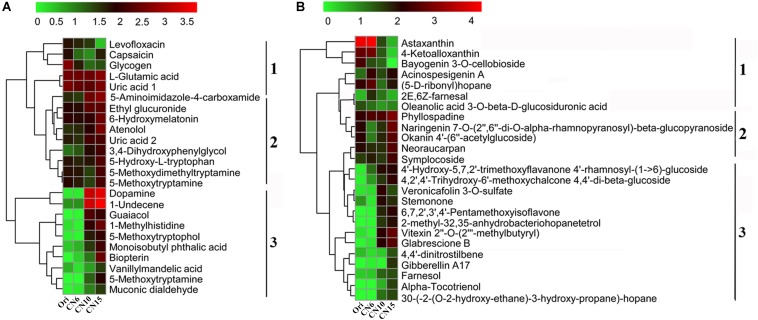
Heatmap was applied to visualize the differences in the composition of metabolites among different groups. **(A)** Metabolites mainly related to benzene, phenols and indoles; **(B)** Metabolites mainly related to polyketides and prenol lipids. Microbial metabolite concentration was scaled with log transformation in the heatmap. 1, cluster 1 represents a significant down-regulated at CN10 and CN15, compared to those at CN6; 2, cluster 2 represents a slightly up-regulated at CN10 and a significantly increased at CN15, compared to those at CN6; 3, cluster 3 represents those with low concentrations in the groups Ori (Day 0) and CN6, but they have high concentrations in the groups CN10 or CN15.

To evaluate the metabolic consequences of increasing C/N ratio, the intestinal samples from the four groups were subjected to a metabolic profiling analysis based on LC-MS analysis. A total of 549 metabolites with significant differences among different groups were identified, including 274 differential metabolites between the groups Ori and CN6, 84 differential metabolites between the groups CN6 and CN10, 313 differential metabolites between the groups CN6 and CN15, and 181 differential metabolites between the groups CN10 and 15 (Figure 6A and Supplementary Table S2). Principle component analysis (PCA) showed that the composition of metabolites among different groups were remarkablely different (Figure 6B). To identify the key metabolites for characterizing the roles of increasing C/N ratio, the top 49 significant metabolites including 13 polyketides, 12 prenol lipids, 11 organoheterocyclic compounds, 7 benzenoids (including 2 benzene derivatives and 5 phenols), 2 carboxylic acid derivatives, 2 organooxygen compounds, 1 hydrocarbon and 1 organooxygen compounds, were divided into three clusters according to their concentrations in different groups (Figure 7). The cluster 1 included 12 metabolites that were significantly down-regulated in the high C/N ratio levels compared to those in CN6. For example, the concentrations of levofloxacin and glycogen were decreased by 57.5–95.2% at CN10 and CN15, compared to those at CN6 (Figure 7A). The astaxanthin, 4-ketoalloxanthin and bayogenin 3-O-cellobioside were abundantly accumulated in the groups Ori and CN6, while they were significantly reduced in the group CN10 and almost disappeared in the group CN15 (Figure 7B). The 14 metabolites in the cluster 2 were slightly up-regulated in the group CN10 and significantly increased in the group CN15, compared to those in the group CN6, such as 6-hydroxymelatonin, atenolol and neoraucarpan. The cluster 3 consisted of 23 metabolites, which had low concentrations in the groups Ori and CN6, while they were amassed by increasing the C/N ratio, especially for the metabolites of dopamine, 1-undecene, biopterin, glabrescione B and gibberellin A17 (Figure 7 and Supplementary Table S2).

### Relations Between OTUs and Metabolites

**FIGURE 8:**
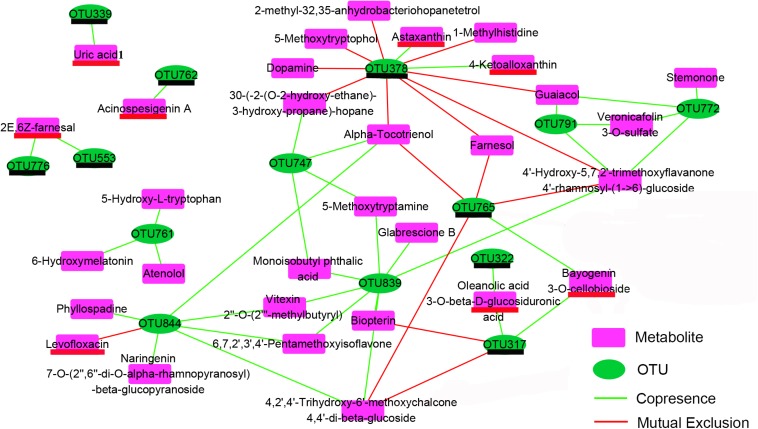
Co-occurrence network depend on the Pearson correlation characterizes the significant correlations between differential OTUs (Figure 3) and differential metabolites (Figure 7) in shrimp intestines. The OTUs underlined in black color belong to the Cluster 3 in Figure 3; the metabolites underlined in red color belong to the Cluster 1 in the Figure 7.

The co-occurrence network characterized the significant correlations between metabolically active bacterial OTUs (Figure 3) and kinds of secondary metabolites (Figure 7) in shrimp intestines (Figure 8). The OTUs belonging to Rhodobacteraceae seemed to play a two-faced role in linking these secondary metabolites. For example, the OTU378 and OTU765 were negatively correlated with 9 and 4 secondary metabolites, respectively, which were mainly involved in phenols and prenol lipids; while the OTU844 was positively related to 5 polyketides, and 1 prenol lipid, and the OTU761 were positively linked with atenolol, 6-hydroxymelatonin, and 5-hydroxy-L-tryptophan. Notably, the OTU378, 765, and 844 were all correlated with alpha-tocotrienol (Figure 8). The OTU747 from Alteromonadanceae was positively related to benzene and indole derivatives and 2 prenol lipids, and three OTUs from Actinobacteria (OTU772, 791, and 839) mainly played a positive role in driving the accumulation of polyketides and indoles, especially the OTU839. In addition, the OTU772 and 791 seemed to play an opposed role with OTU378 and 765; while OTU839 had a synergistic effect with OTU844 and 747. Cyanobacteria OTU322 might promote the accumulation of another Cyanobacteria OTU317 through the secondary metabolite of oleanolic acid 3-O-bata-D-glucosiduronic, and the accumulation of OTU317 would restrain the production of biopterin and 4,2′,4′-Trihydroxy-6′-methoxychalcone 4,4′-di-beta-glucoside (TMDG). Moreover, the OTU765 also positively affected the OTU317 by the metabolite of bayogenin 3-O-cellobioside, and then reduced the production of TMDG (Figure 8).

## Discussion

### High C/N Ratio Promoted the Shrimp Growth and Bioflocs Formation

Biofloc technology through increasing the C/N ratio of the aquaculture water has recently been widely used in intensive shrimp aquaculture. It has been reported that regulating C/N ratio can improve water quality and promote shrimp growth and health by forming the microbial biofloc (Xu and Pan, 2013; Julie et al., 2014). In this study, the biofloc amount at CN10 and CN15 were 3.3-, 4.5-fold higher than that at CN6, and correspondingly the shrimp length and weight were significantly higher at the high C/N ratio levels, especially at CN15 (Figure 1), indicating that increasing C/N ratio is beneficial for biofloc accumulation and shrimp growth. It was similar with that the high C/N ratio helps to form a large of bioflocs consisting of abundant protein and bioactive compounds (Emerenciano et al., 2014), which not only provide a supplemental food source but also enhance the immune response, antioxidant status and digestive enzyme activity of shrimp, thus improving growth performance, disease resistance and feed utilization of the cultured shrimp (Xu and Pan, 2013; Julie et al., 2014). In addition, the bioflocs included a number of algae and heterotrophic bacteria, which can improve water quality by stimulating the process of inorganic nitrogen assimilation, and thus providing a good environment for the growth of aquatic animals (Schveitzer et al., 2013; Wei et al., 2016).

### High C/N Ratio Shifted the Intestinal Microbiota of Shrimp

Intestinal microbial community plays an important role in maintaining intestinal micro-ecological balance because of its close relationship with nutrient acquisition (Sullam et al., 2015) and pathogen defense (Yan et al., 2014), which are necessary for the growth and health of the shrimp. It has been reported that sugars feeding can selectively impact beneficial microbes and promote intestinal health (Rastall et al., 2015). In this study, the bacterial α-diversity decreased as the input of C/N ratio increased, and the bacterial community compositions of groups CN10 and CN15 were significantly different from that of group CN6 (Figure 2 and Table 1). This was similar with that the application of exogenous glucose could simplify the microbial community and increase the abundance of some specific bacterial groups, such as the bacteria affiliated with the families Rhodobacteraceae and Alteromonadaceae (Gómez-Consarnau et al., 2012; Goldford et al., 2018). Members of the Rhodobacteraceae are deeply involved in carbon/sulfur cycling and symbiosis with aquatic animals (Pujalte et al., 2014). The present study found that the higher relative abundance of Rhodobacteraceae in the gut of shrimp was found at the CN10 and CN15, compared to that at CN6 (Figure 2C), indicating that increasing C/N ratio was positively related to the enrichment of Rhodobacteraceae. In the top 40 abundant OTUs, three *Roseobacter* clade members from Rhodobacteraceae (*Dinoroseobacter*, *Ruegeria* and *Roseobacter* clade CHAB-I-5 lineage) enriched in the groups CN10 and CN15 were mainly involved in producing various beneficial chemicals (Sañudo-Wilhelmy et al., 2014; Sonnenschein et al., 2017), and thus these taxa can be regarded as the potential beneficial bacteria. It has been reported that *Ruegeria* can produce a broad-spectrum antibiotic (TDA) and has been thought as a potential probiotic in aquaculture that can limit the growth of pathogens, such as *Vibrio anguillarum* (Sonnenschein et al., 2017). *Dinoroseobacter*, is reported to have a symbiotic relationship with algae through producing vitamins (B12), which are also necessary for growth of aquatic animals (Sañudo-Wilhelmy et al., 2014). In addition, the *Paracoccus* from Rhodobacteraceae enriched in the groups CN10 and CN15 was mainly related to catalyze denitrification, degrade various organic pollutants, and inhibit the growth of *Vibrio splendidus* in sea cucumbers (Liu et al., 2012; Yan et al., 2014; Wang et al., 2018). Members of the Alteromonadaceae contain various genes related to organic degradation and secondary metabolite synthesis (López-Pérez and Rodriguez-Valera, 2014), and these taxa have indeed shown extensive degradative properties, which may be beneficial for the growth of host. The relative abundance of Actinobacteria were up-regulated by increasing the C/N ratio input (Figures 3, 4). Actinobacteria has been well known as an important source of bioactive natural products, which show a variety of biological properties including antimicrobial, anticancer, antiviral, insecticidal, and enzyme inhibitory activities (Manivasagan et al., 2014). In addition, the changes of relative abundance in Actinobacteria, Alteromonadaceae and Rhodobacteraceae may also be partially due to the difference of them in rearing water caused by increasing C/N ratio input (Gómez-Consarnau et al., 2012; Shang et al., 2018). Adding an exogenous carbon source can significantly change the microbial community of the rearing water (Panigrahi et al., 2018). Aquatic animals are highly exposed to microbial loads in the aquatic environment, and this closer contact with the surrounding water very likely influences microbial colonization in gut (Giatsis et al., 2015).

Exogenous carbon source application enormously induced the growth of heterotrophic bacteria (Avnimelech, 1999), which may reduce the diversity of intestinal microbiota by restraining the growth of some bacteria, like pathogenic bacteria and autotrophic microbe. In this study, the OTUs belonging to Cyanobacteria, Mycoplasmataceae and *Vibrio* were overrepresented in the group CN6 (Figures 3, 5), which had the worst growth performance of shrimp (Figure 1), and thus these taxa might be regarded as the detrimental bacteria. Cyanobacteria species have been reported to have detrimental influences on aquatic ecosystems through altering trophic structure and functionality, and reducing the deoxygenation and quality of water (Catherine et al., 2013). Blooms of Cyanobacteria may release a number of toxins, such as the hepatotoxic microcystins, nodularins and cylindrospermopsins, the neurotoxic saxitoxins, anatoxin-a and homoanatoxin-a and dermatotoxins, which can cause rapid death in animals (Neilan et al., 2013). The high C/N ratio application significantly reduced the accumulation of OTUs belonging to Cyanobacteria, especially the OTU317, 322, and 533, further indicating the good shrimp growth at CN10 and CN15 was partially related to the decrease in the relative abundance of Cyanobacteria (Figures 1, 5). Some members of Mycoplasmataceae, like *Mycoplasmas* and *Ureaplasmas* have been identified as human pathogens and may cause severe systemic disease of respiratory and urogenital tracts in susceptible hosts (Waites et al., 2013). Vibriosis has been regarded as one of the most prevalent disease caused by certain *Vibrio* spp., such as *V. harveyi*, *V. parahaemolyticus*, *V. alginolyticus*, *V. anguillarum*, *V. vulnificus*, and *V. splendidus* etc., in shrimp and other aquaculture animals (Chatterjee and Haldar, 2012). The high abundance of *Vibrio* in aquaculture systems is positively related with shrimp mortality (Joshi et al., 2014). Mycoplasmataceae OTU553 and *Vibrio* OTU569 were detected in large quantities at CN6; while the numbers of them were markedly reduced by increasing the C/N ratio input, especially at CN15. These results may further explain the better growth of shrimp at CN10 and CN15 than that at CN6 (Figure 1). In addition, the high abundance of Actinobacteria, Alteromonadaceae and Rhodobacteraceae induced by increasing C/N ratio could built a beneficial bacterial community in the intestinal tract of shrimp, and thus suppressing the survival of pathogenic bacteria (Figures 4, 5; Shang et al., 2018).

### Bioactive Metabolites Highly Correlated With Microbial Community

The crosstalk between the intestinal microbiota and its host depends in partly on the production of metabolites, which have a great influence on host physiology (Levy et al., 2017). In this study, totally 14 OTUs showed significant (*P* < 0.05) correlation with 33 metabolites mainly including flavonoids, benzenoids, indole derivatives and prenol lipids through the network analysis (Figure 8). Flavonoids possess potentially exploitable activities, including direct antibacterial activity, synergism with antibiotics, and suppression of bacterial virulence (Cushnie and Lamb, 2011). Benzenoids usually exhibit potent inhibition against superoxide production and have well anti-inflammatory activities (Chen et al., 2007). Indole is the metabolite of amino acid tryptophan, which has recently been proven to participate in the quorum sensing and biofilm formation (Hu et al., 2010). Prenol lipids were known as the precursors of polyterpenes and vitamins (Sharma et al., 2017; Raj and Priya, 2019). The chemicals of polyterpene have the antibacterial activity against various pathogenic bacteria, and vitamins were the important nutrition components for the growth of plants and animals (Heydarizadeh et al., 2014; Combs and Mcclung, 2016). Interestingly, the Rhodobacteraceae OTU761 and 844 upregulated by adding exogenous sucrose were main positively related to a number of indole derivatives and flavonoids, respectively, while another two downregulated Rhodobacteraceae OTUs (OTU378 and 765) were mainly negatively corelated with prenol lipids (Figure 8 and Supplementary Table S3). These results indicated that Rhodobacteraceae may have the positive and negative effects on the growth of shrimp, although it has been widely reported as a potential source of probiotics. Moreover, Actinobactria OTU839 and Alteromonadaceae OTU747 were also positively associated with various flavonoids, benzenoids and indole derivatives (Figure 8). Therefore, it was speculated that the exogenous sucrose application may promote the growth of shrimp through the following two reasons. First, exogenous sucrose application induces the growth of certain bacteria, which can secret various bioactive metabolites to inhibit the growth of Mycoplasmataceae (OTU553) and *Vibrio* (OTU569) (Figure 5). Second, exogenous sucrose application can inhibit the growth of certain bacteria to accumulate the chemicals of prenol lipids, and in turn the accumulation of prenol lipids not only provides the nutrients for the growth of beneficial bacteria and shrimp but also further limits the growth of some detrimental bacteria. In addition, some species of Rhodobacteraceae can produce the astaxanthin (Pujalte et al., 2014), which are positively related to the OTU378, indicating that the OTU378 may induce the produce of astaxanthin (Figure 8). Two Cyanobacteria OTUs (OTU322 and 317) can positively affect the abundance of OTU765 through influencing the production of some metabolites. It is consistent with that the abundance of microalgae is significantly decreased due to the increase of heterotrophic bacteria by adding exogenous sucrose, and the abundance of Cyanobacteria and Rhodobacteraceae can be simultaneously decreased by removing the microalgae (Bulleri et al., 2018).

## Conclusion

The present study showed that increasing the C/N ratios by adding sucrose can significantly promote the growth of shrimp and increase the bioflocs volume in the aquaculture systems. The better growth performance of shrimp at CN10 and CN15 might be closely associated with the significant change of the intestinal microbiota and various metabolites. The high C/N ratio input increased the relative abundance of some potential beneficial bacteria, like Actinobacteria, Rhodobacteraceae (mainly consist of *Roseobacter* and *Paracoccus* groups), Alteromonadaceae, and inhibited the growth of detrimental bacteria, such as Cyanobacteria, Mycoplasmataceae and *Vibrio*. The increase of potential beneficial bacteria would promote the accumulation of various bioactive metabolites, like flavonoids and benzenoids, which can further inhibit the growth of detrimental bacteria. In addition, the increase of C/N ratio also reduce the abundance of certain Rhodobacteraceae, and the decrease of them helped to the production of prenol lipids, which may further improve the growth of potential beneficial bacteria and shrimp.

## Data Availability Statement

All sequencing data have been deposited (PRJCA002328) in the Genome Sequence Archive in the BIG Data Center, Chinese Academy of Sciences under accession codes CRA002442 for bacterial 16S rRNA gene sequencing data that are publicly accessible at http://bigd.big.ac.cn/gsa.

## Ethics Statement

All animal experiments were carried out in accordance with the United Kingdom Animals (Scientific Procedures) Act, 1986, and associated guidelines, EU Directive 2010/63/EU for animal experiments.

## Author Contributions

HG and DZ provided the experimental ideas and design of this study. LH, SH, SW, CC, and XH did the experiments. WL, YYZ, and YJZ helped to analyze the data. HG and SH wrote the manuscript. LH and DZ revised the manuscript. All authors approved the final manuscript.

## Conflict of Interest

The authors declare that the research was conducted in the absence of any commercial or financial relationships that could be construed as a potential conflict of interest.

## Footnotes

1 http://www.hmdb.ca/

2 http://www.lipidmaps.org/
