# Supplementary material for: Effects of Carbon/Nitrogen Ratio on Growth, Intestinal Microbiota and Metabolome of Shrimp (Litopenaeus vannamei)
Source: Front Microbiol. 2020 Apr 15;11:652. doi: 10.3389/fmicb.2020.00652 (PMC7176362; doi:10.3389/fmicb.2020.00652)
Supplement: Supplementary file 1 [file Data_Sheet_1.doc]

**Supplemental Data Summary**

| **OTU No.** | **Taxa** |
| --- | --- |
| OTU378 | Proteobacteria; Alphaproteobacteria; Rhodobacterales; Rhodobacteraceae; g__unclassified |
| OTU660 | Proteobacteria; Alphaproteobacteria; Rhodobacterales; Rhodobacteraceae; g__*Dinoroseobacter* |
| OTU761 | Proteobacteria; Alphaproteobacteria; Rhodobacterales; f__Rhodobacteraceae |
| OTU765 | Proteobacteria; Alphaproteobacteria; Rhodobacterales; Rhodobacteraceae; g__*Tropicimonas* |
| OTU840 | Proteobacteria; Alphaproteobacteria; Rhodobacterales; Rhodobacteraceae; g__unclassified |
| OTU844 | Proteobacteria; Alphaproteobacteria; Rhodobacterales; Rhodobacteraceae; g__*Paracoccus* |
| OTU317 | Cyanobacteria; norank; norank; norank; norank; s__*Picochlorum*_sp._SENEW3 |
| OTU322 | Cyanobacteria; norank; norank; norank; g__Picochlorum eukaryotum |
| OTU533 | Cyanobacteria; norank; norank; norank; g__Picochlorum eukaryotum |
| OTU772 | Actinobacteria; Actinobacteria; Propionibacteriales; Propionibacteriaceae; g__Tessaracoccus |
| OTU791 | Actinobacteria; Actinobacteria; Micrococcales; Microbacteriaceae; g__Microbacterium |
| OTU839 | Actinobacteria; Actinobacteria; Micrococcales; Demequinaceae; g__unclassified |
| OTU553 | Tenericutes; Mollicutes; Mycoplasmatales; Mycoplasmataceae; g__Candidatus_Bacilloplasma |
| OTU747 | Proteobacteria; Gammaproteobacteria; Alteromonadales; Alteromonadaceae; g__unclassified |
| OTU569 | Proteobacteria; Gammaproteobacteria; Vibrionales; Vibrionaceae; g__*Vibrio* |

**Table S1** The detailed information of indicator taxa (OTU) of the shrimp intestinal microbial community.


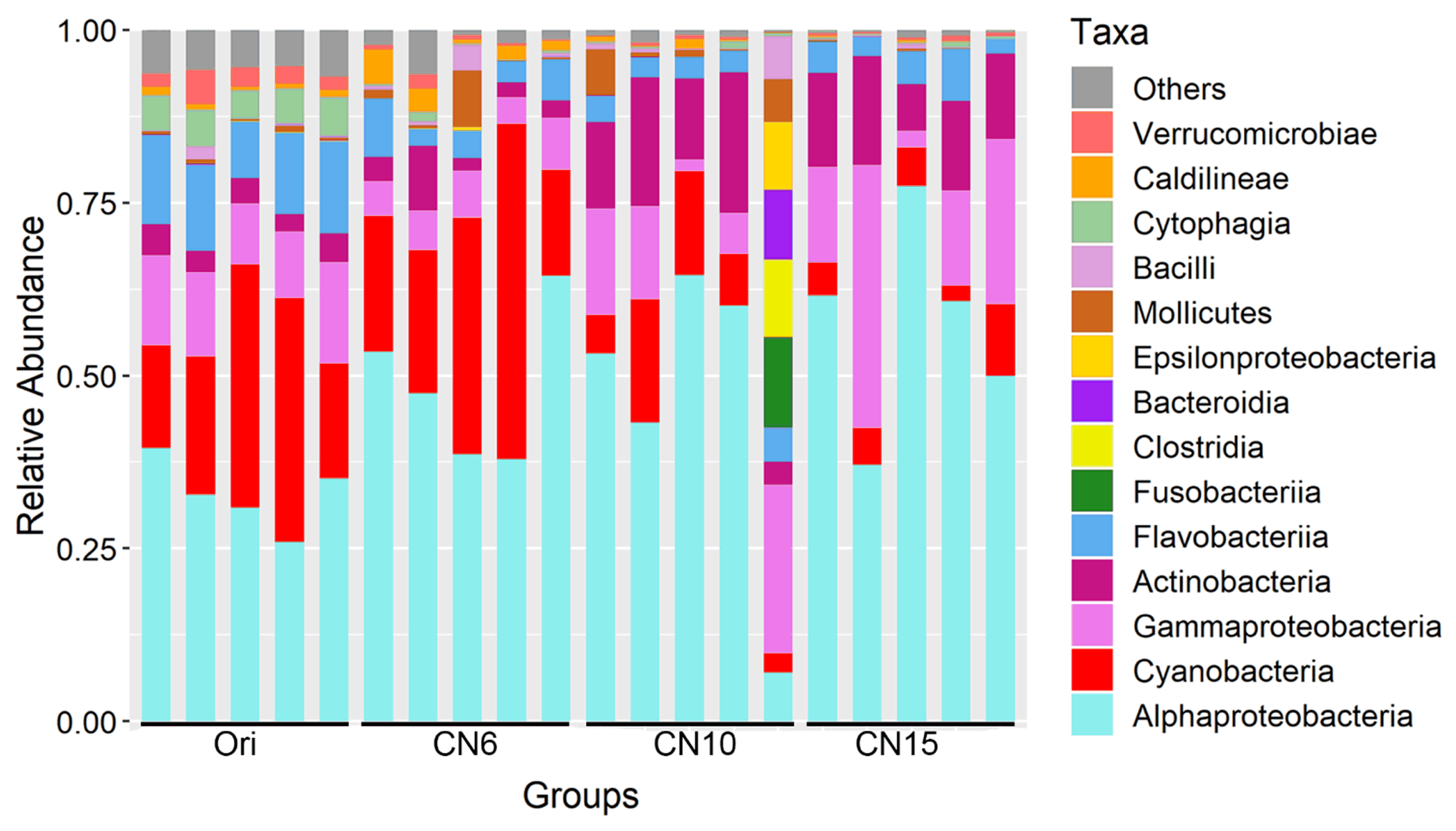


**Figure S1** Relative abundances of the dominant intestinal bacteria of *L. vannamei* at the classes level (relative abundance > 1%).
